# Supplementary material for: Revisiting genomes of non-model species with long reads yields new insights into their biology and evolution
Source: Front Genet. 2024 Feb 7;15:1308527. doi: 10.3389/fgene.2024.1308527 (PMC10879605; doi:10.3389/fgene.2024.1308527)
Supplement: Supplementary file 1 [file DataSheet1.pdf]

Supplementary Material

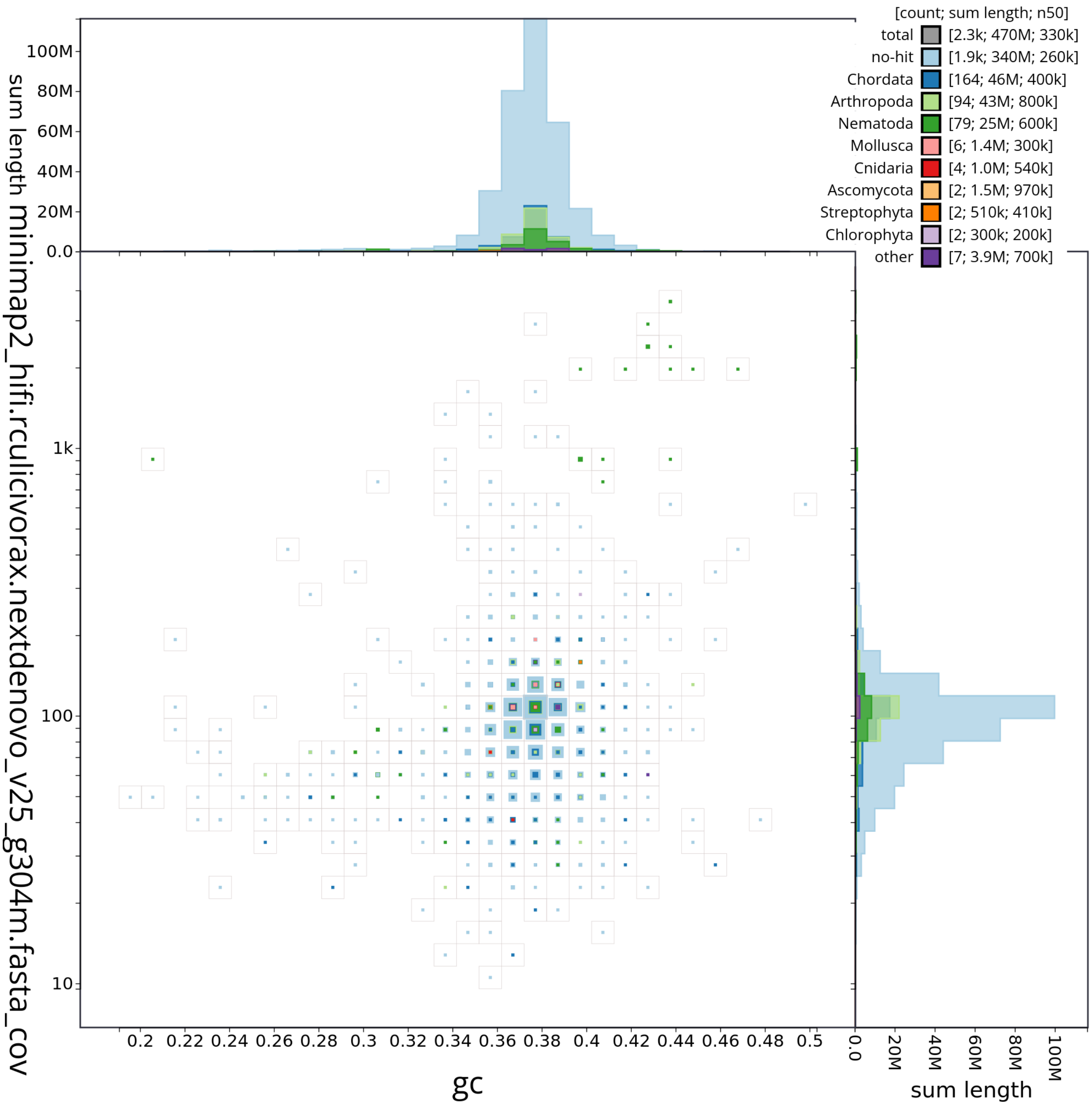

Figure S1. Example of Blobtools analysis for the NextDenovo PacBio HiFi assembly of *Romanomermis culicivorax*.

Table S1. Statistics of the PacBio HiFi datasets.

Species	Size	N50
Romanomermis culicivorax	37,542,673,078	12,613
Panagrolaimus sp. PS1159	29,159,296,422	15,797

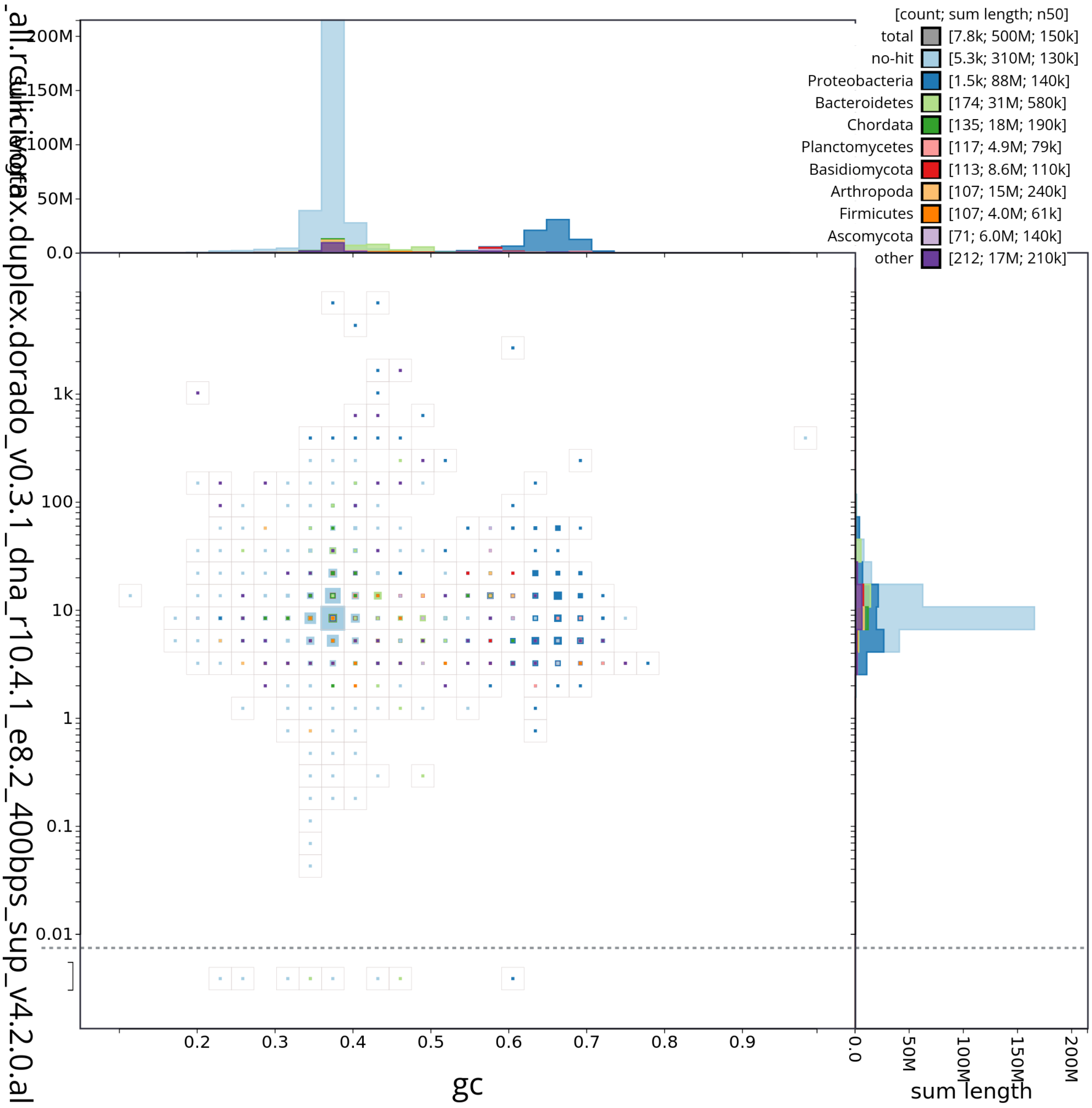

Figure S2. Example of Blobtools analysis for the Flye Nanopore assembly of *Romanomermis culicivorax*.

Table S2. Statistics of the Nanopore datasets.

Species	Quality threshold	Size	N50	Largest
Romanomermis culicivorax	0	5,697,172,871	15,936	540,992
	10	5,021,948,640	15,980	218,858
	15	3,943,100,749	15,551	218,858
	20	979,904,894	15,886	115,632
Panagrolaimus sp. PS1159	0	10,696,877,601	33,428	914,599
	10	9,344,589,639	33,814	205,535
	15	8,280,860,091	33,820	205,535
	20	4,718,164,203	34,667	173,662

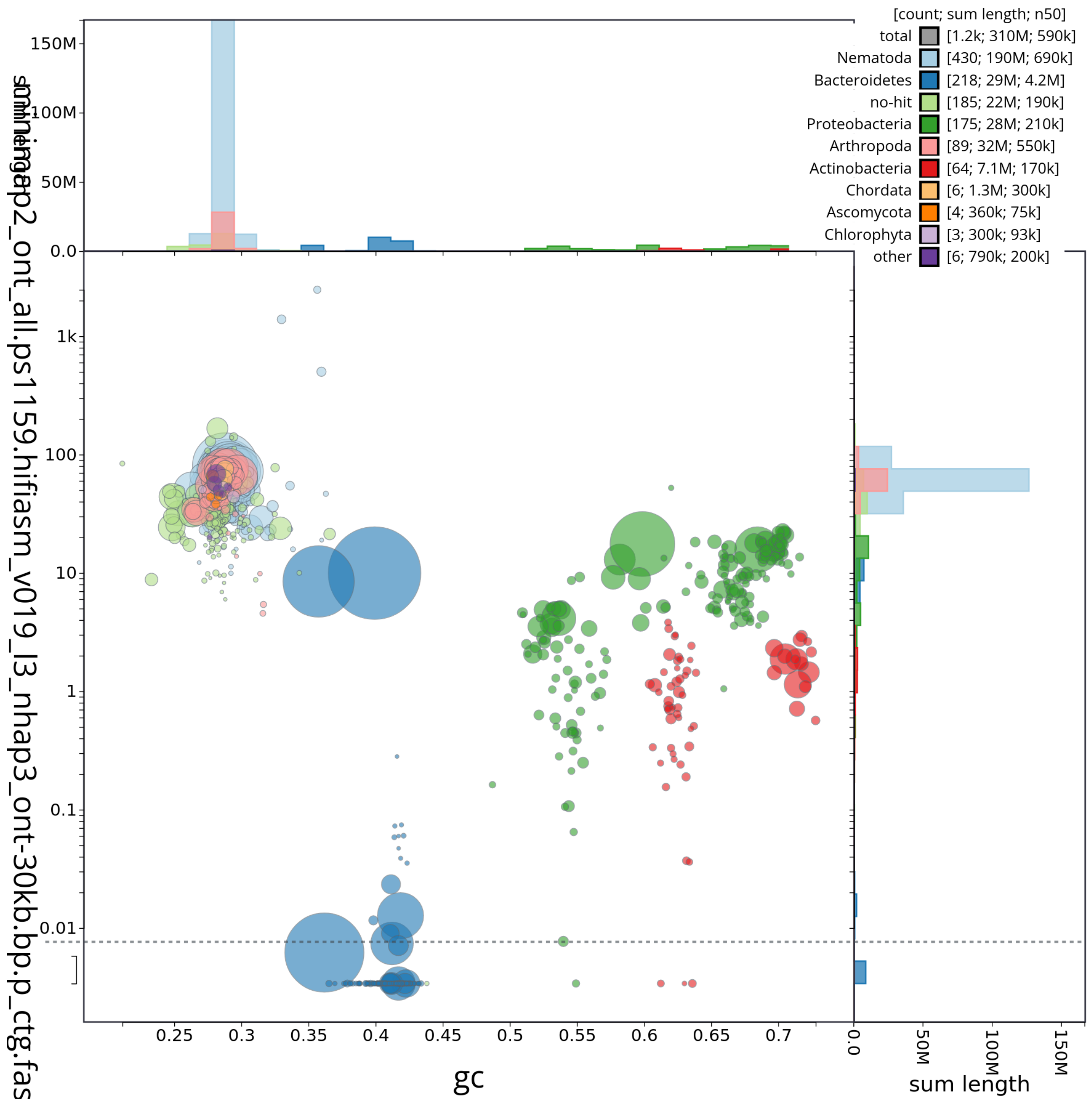

Figure S3. Example of Blobtools analysis for the hifiasm PacBio HiFi + Nanopore assembly of *Panagrolaimus* sp. PS1159.

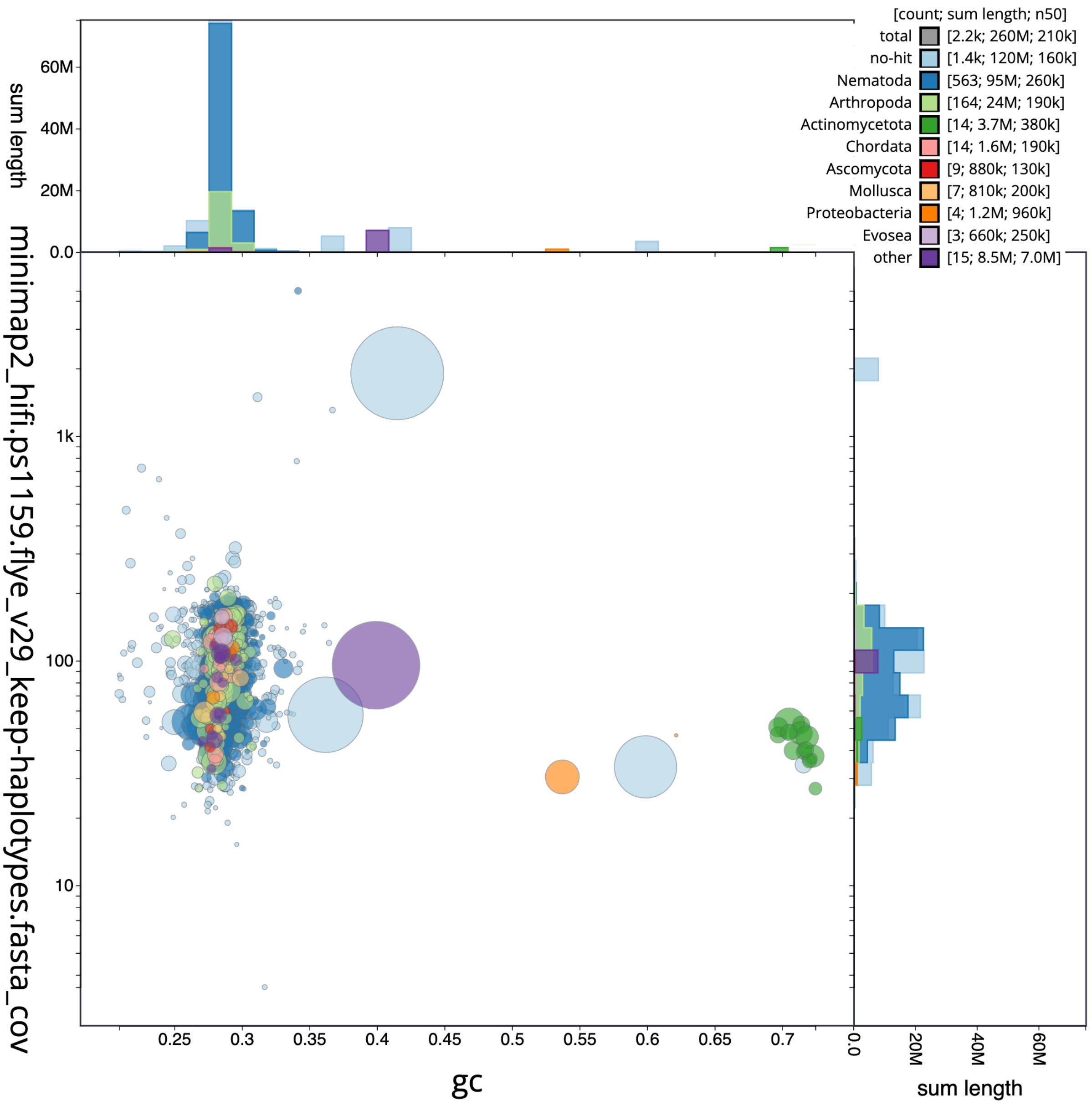

Figure S4. Example of Blobtools analysis for the Flye Nanopore assembly of *Panagrolaimus* sp. PS1159.

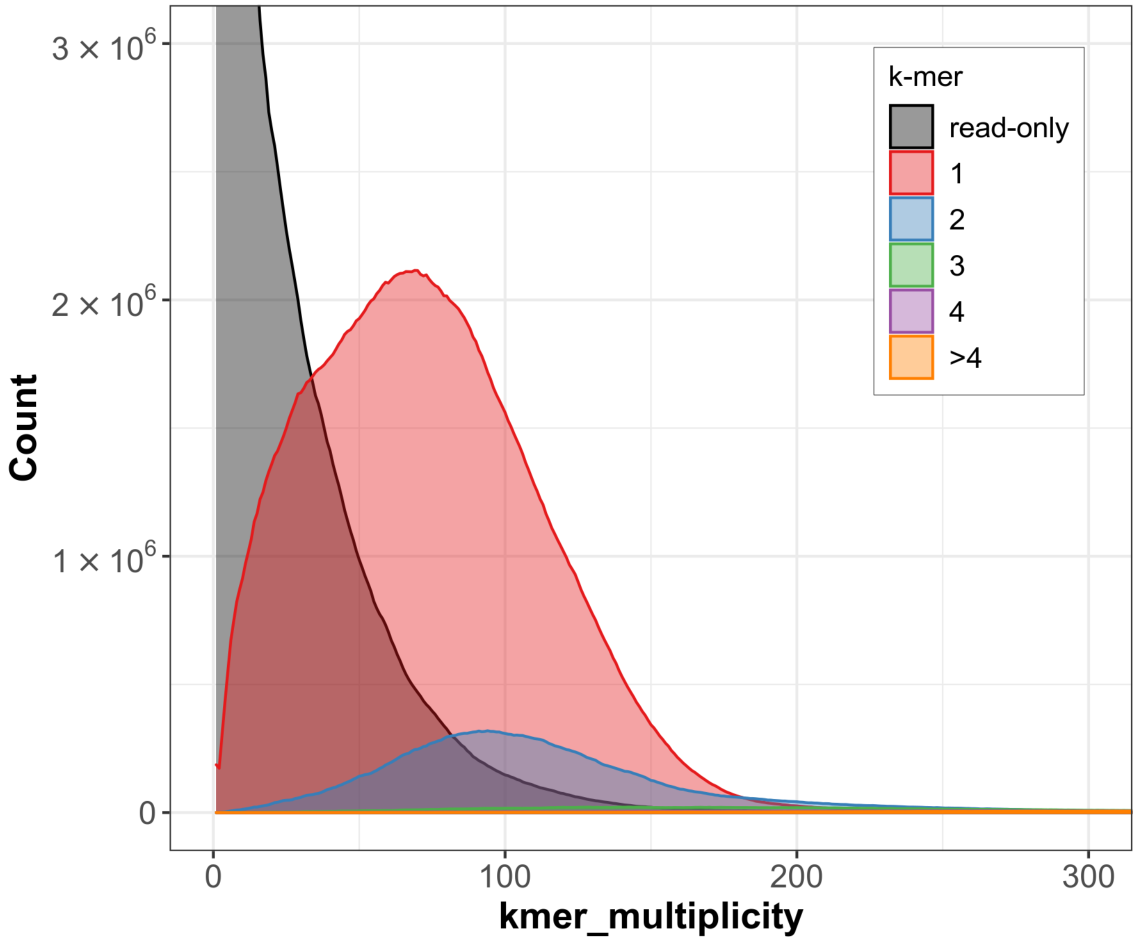

Figure S5. Merqury analysis of the final assembly of *Romanomermis culicivorax*.

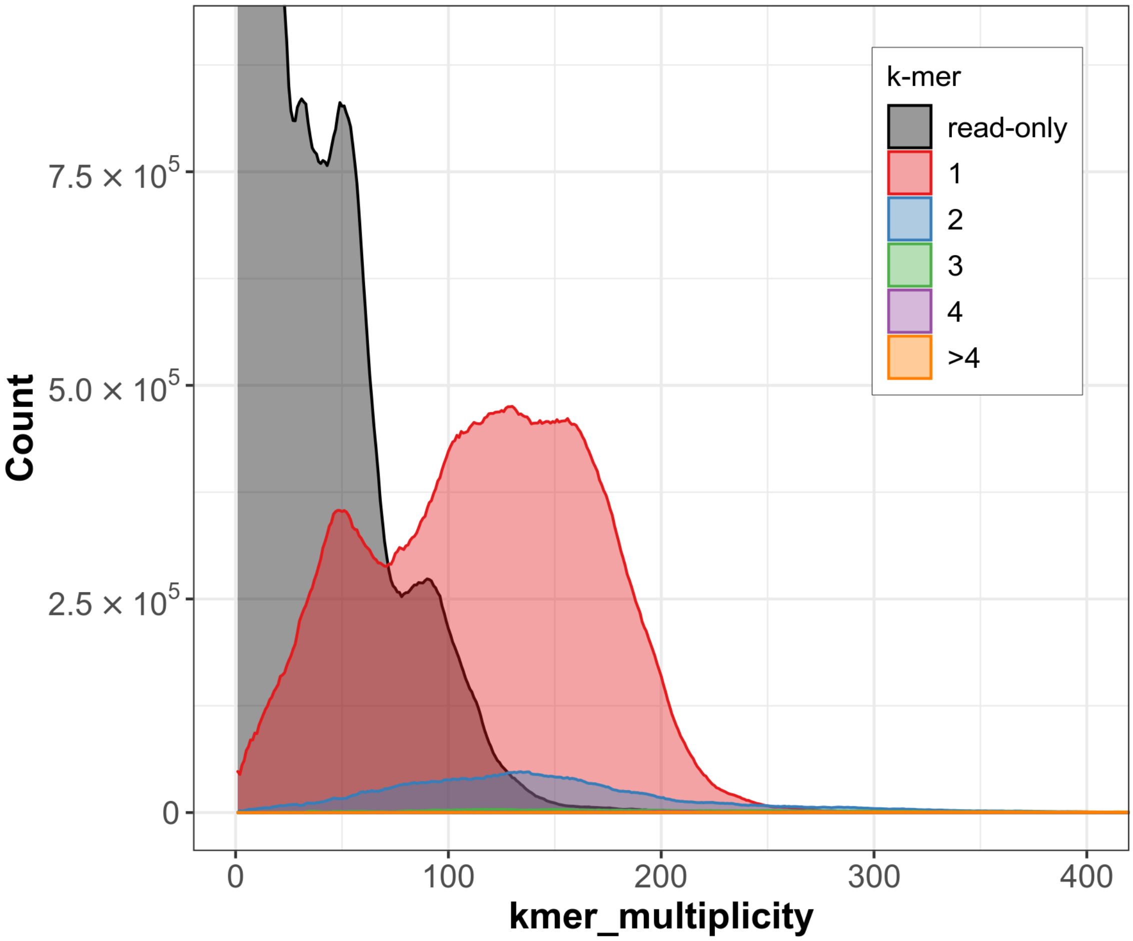

Figure S6. Merqury analysis of the final assembly of *Panagrolaimus* sp. PS1159.

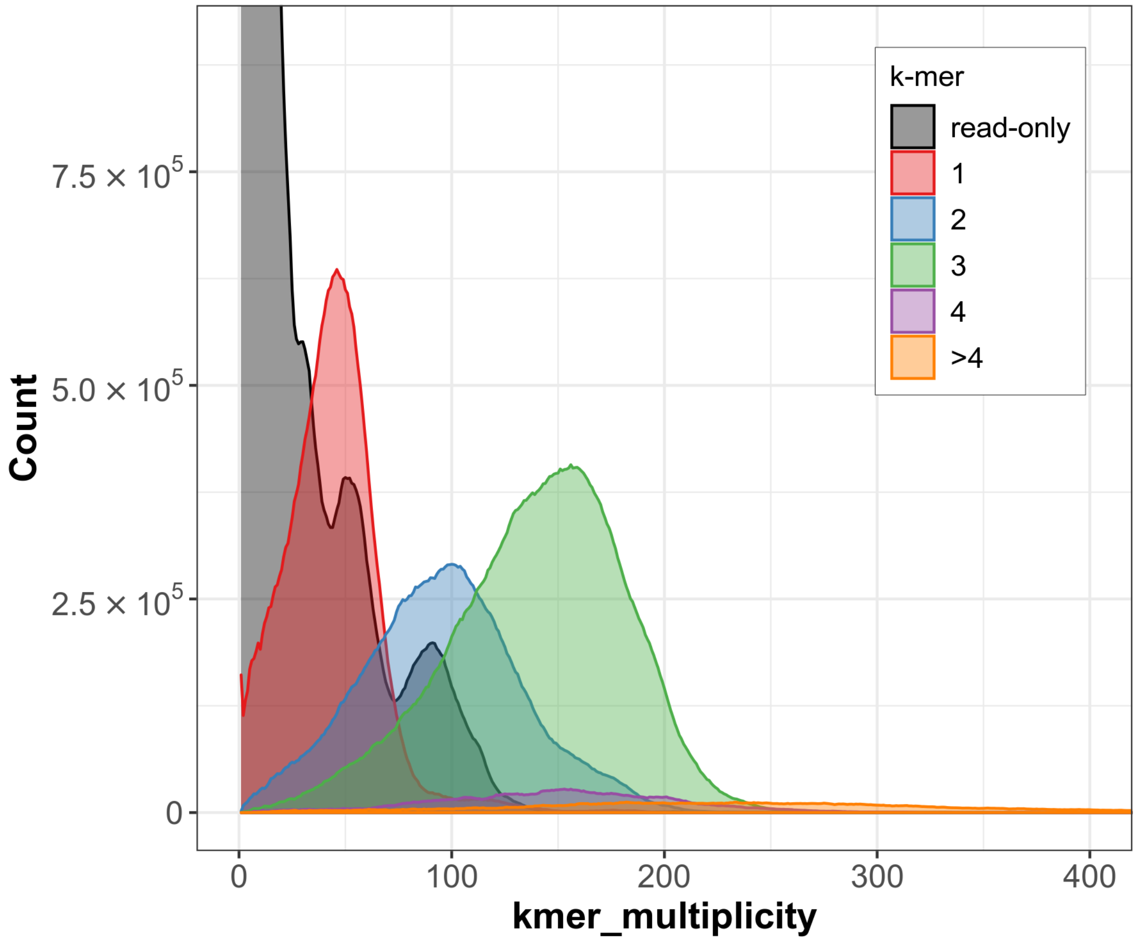

Figure S7. Merqury analysis of the phased assembly of *Panagrolaimus* sp. PS1159.

Species	Assembly	Category	Count
Romanomermis culicivorax	v1	Unclassified	36,611,241
		I-LTR	2,623,729
		I-DIRS	380,102
		I-Penelope	198,523
		I-LINE	987,323
		II-helitron	8,326,702
		II-polinton	444,560
		II-TIR	81,385,493
	v2	Unclassified	47,999,208
		I-LTR	103,047,244
		I-DIRS	71,775
		I-Penelope	0
		I-LINE	881,202
		II-helitron	7,860,451
		II-polinton	6,478,667
		II-TIR	123,251,468
Panagrolaimus sp. PS1159	v1	Unclassified	3,813,266
		I-LTR	59,899
		I-DIRS	0
		I-Penelope	0
		I-LINE	6,942
		II-helitron	5,224
		II-polinton	0
		II-TIR	475
	v2	Unclassified	7,442,799
		I-LTR	3,983,941
		I-DIRS	0
		I-Penelope	143,146
		I-LINE	44,333
		II-helitron	380,276
		II-polinton	18,632
		II-TIR	4,864,827

Table S3. Transposable elements counts.
